# A Hypothetical Model Suggesting Some Possible Ways That the Progesterone Receptor May Be Involved in Cancer Proliferation

**DOI:** 10.3390/ijms222212351

**Published:** 2021-11-16

**Authors:** Jerome H. Check, Diane L. Check

**Affiliations:** 1 Department of Obstetrics and Gynecology, Division of Reproductive Endocrinology & Infertility, Cooper Medical School of Rowan University, Camden, NJ 08103, USA; 2 Cooper Institute for Reproductive Hormonal Disorders, P.C., Mt. Laurel, NJ 08054, USA; jerryccivf@gmail.com

**Keywords:** progesterone receptors, progesterone receptor modulators, malignant tumors, tumor microenvironment, cellular immunity, natural killer cells, mifepristone, fetal-placental unit

## Abstract

Cancer and the fetal-placental semi-allograft share certain characteristics, e.g., rapid proliferation, the capacity to invade normal tissue, and, related to the presence of antigens foreign to the host, the need to evade immune surveillance. Many present-day methods to treat cancer use drugs that can block a key molecule that is important for one or more of these characteristics and thus reduce side effects. The ideal molecule would be one that is essential for both the survival of the fetus and malignant tumor, but not needed for normal cells. There is a potential suitable candidate, the progesterone induced blocking factor (PIBF). The parent 90 kilodalton (kDa) form seems to be required for cell-cycle regulation, required by both the fetal-placental unit and malignant tumors. The parent form may be converted to splice variants that help both the fetus and tumors escape immune surveillance, especially in the fetal and tumor microenvironment. Evidence suggests that membrane progesterone receptors are involved in PIBF production, and indeed there has been anecdotal evidence that progesterone receptor antagonists, e.g., mifepristone, can significantly improve longevity and quality of life, with few side effects.

## 1. Knowledge of Transplant Immunology Has Led to Some Effective Anticancer Therapies

### Autologous Immunotherapy

Somatic mutations in cancer cells lead to the production of unique antigens that are present on the cancer cell surface. These mutations can alter the sequence of proteins and these new antigens create new epitopes [1]. These new epitopes are processed on major histocompatibility complex molecules complex (MHC) molecules. The neo-epitopes are recognized by T-cells and are called neoantigens [2].

These new antigens (previously known as tumor specific transplantation antigen, or tumor associated antigens) can be destroyed by a host immune response, especially by CD8+ T-lymphocytes. The killing of malignant tumor cells requires the recognition by the T-cell receptor (TCR) of specific antigenic peptides presented on the surface of malignant cells by human leukocyte antigens (HLC)-1/Beta 2-microblobulin complex [3]. This process eventually leads to a transduction cascade with the initial final killing process given to cytotoxic T-lymphocytes (CTLs) [3].

The CTLs an natural killer (NK) cells may kill cancer cells directly through degranulation of perforin granules leading to lethal release of perforin or by exocytosis of granzymes [4]. Immune response against malignant tumors can also be achieved indirectly through secretion of various cytokines, e.g., tumor necrosis factor (TNF) alpha or interferon gamma [4].

Documentation of the importance of immune inhibitors by CD8+ T-cells was demonstrated by isolation of tumor specific CTLs from the tumor or the peripheral blood from patients with cancer [5,6,7]. Activation of naïve CD8+ T-cells by antigen presenting cells (APC) involves binding TCR that is associated with CD3 complex, to specific peptide-major histocompatibility complex class I complexes and the interaction of the co-stimulatory molecules CD80/CD86 or LFA3 [8].

The presence of neoantigens in cells that have had malignant transformation may be occurring continuously, but immune surveillance by CTLs prevent malignant tumor formation. However, some mechanism allows some malignant cells to form into a malignant tumor, and most likely, successful transformation requires somehow cloaking these neo-antigens and making them less immunogenic.

Thus, one method to improve immunogenicity of the host was by injecting back into the host a vaccine made of killed tumor cells. This method did have some limited success in thwarting murine cancer growth [9,10,11,12,13,14]. Subsequent attempts were made to make these killed tumor cells more immunogenic [15,16,17,18].

Present day methods that have been used to treat humans with some success involve methods of expanding subsets of mature T-cells in vitro using selective tumor-reactive T-cells and tumor infiltrating lymphocytes. Clonal repopulation of T-cells directed against over-expressed self-derived differentiation antigens in combination with chemotherapy and interleukin (IL)-2 in high doses, or the use of TILs, has been associated with regression of metastatic melanoma [19,20,21]. Unfortunately, this beneficial effect has not been shown to be effective with other solid malignant tumors, partially related to the rare infiltration of CTLs in solid malignant tumors. Furthermore, TILs seem to be inhibited by the high expression of immune inhibitory receptors, e.g., programmed death factor-1 (PD1) or the cytotoxic T lymphocyte-associated molecule-4 (CTLA-4) or other check-point inhibitors [19,20].

A newer autologous-type of immunotherapy is called chimeric antigen-receptor T-cells (CAR-T) immunotherapy. For hematologic malignancies, for which CAR-T therapy is most effective, engineered T-cell strategy utilizes CAR, comprising the antigen-binding domain of an antibody, fused with one or more immunostimulating domains, to activate T-cells once the recognition domain has bound to a target cell. As T-cells are able to recognize tumor antigen-expressing cells in an MHC-independent manner, a single CAR can be used on all patients whose tumor expresses the target antigen (e.g., CD19 or CD20 [22,23,24,25,26]). This therapy is far more effective for hematologic malignancies than solid tumors [23,24,25,26].

## 2. The Developing of Malignant Tumors with Neoantigens May Utilize Systems That Provide the Body a Mechanism to Control Self-Tolerance Check-Point Inhibitors

### 2.1. Cytotoxic T-Lymphocyte Associated Molecule-4 (CTLA-4)

Treatment aimed to suppress cytotoxic T-lymphocyte-associated molecule-4, an inhibitory molecule expressed by activated T-cells with blocking antibody against CTLA-4 in patients with tumors that have a high number of neo-antigens, have shown clinical benefit in some malignancies [27,28]. One such anti-CTLA-4 check-point inhibitors is called ipilimumab. Cytotoxic T-lymphocyte associated molecule-4 mediates immune suppression by indirectly diminishing signaling through the co-stimulatory receptor CD28. By limiting CD28 mediated signaling during antigen presentation, CTLA-4 increases the activating threshold of T-cells, thus reducing immune responses to weak antigens, e.g., self and tumor antigens [27,28]. Thus, inhibiting CTLA-4 with ipilimumab can enhance a T-cell mediated attack of certain tumors.

### 2.2. Programmed Cell Death (PD-1) and Programmed Cell Death Ligand 1 and Ligand 2

PD-1 is a protein on the surface of activated T-cells. If another molecule called PD-L1 or PD-L2, binds to PD-1, the T-cell becomes inactive. Thus, to re-kindle the T-cell attacking ability against foreign antigens, blocking either PD-1 or PD-L1 (or PD-L2), reactivates the T-cell. Drugs, e.g., nivolumab, which is an antibody directed against PD-1, allows the T-cell to once again react against antigens in the tumor, and thus inhibit growth and by inducing death. Another commonly used anti-PD-1 drug is called pembrolizumab [29]. Combining the anti-CTLA-4 blocking antibody drug ipilimumab with nivolumab increased the effectiveness of nivolumab [29].

However, because the removal of check-point inhibitors to activate T-cells can also direct an immune response against self-antigens, the development of autoimmune disorders is not uncommon and can be life-threatening. Nevertheless, in general, immunotherapy based on inhibiting check-point inhibitors is more effective and less toxic than standard chemotherapy. Thus, check-point inhibitors, along with targeted chemotherapy, is slowly replacing standard chemotherapy as first-line treatment for metastatic disease.

## 3. Survival of the Fetal-Placental Unit and Malignant Tumors

### 3.1. Similarities between Trophoblast and Cancer Cells

Both trophoblast cells and cancer cells must have the ability to invade tissues. Both trophoblast cells and cancer cells have a high proliferation rate. Furthermore, they both have the ability to promote angiogenesis for survival despite both starting out with a lack of vascularity leading to hypoxia [30,31]. Furthermore, both the fetal-placental unit and the tumor have the need to evade immune surveillance and thus have to actively modulate the immune response [31,32].

### 3.2. The Role of Distinct Lymphocyte Cell Population in Regulating Feto-Maternal Tolerance and Tolerance to Malignant Tumors

It is becoming clearer that both in transplantation immunology and immunology of pregnancy, immune tolerance requires a balance of different lymphoid cell populations in the placental and tumor tissue, per se. The cells that must be balanced are regulatory T-cells (Treg), effector T-cells (Teff), T-helper cells (TH17 and TH1 cells), and costimulatory pathways (e.g., PD1-PDL1 negative costimulatory pathways) [33,34,35]. Tregs are important in suppressing cytotoxic T-cells from attacking the fetal semi-allograft and the tumor cells with neoantigens. Simply stated, when there is a balance favoring TH1 and TH17 cells, there is a shift toward immune rejection. In contrast, when there is a shift toward an increase in Treg cells, there is immune tolerance. Check-point inhibitors may serve to help the shift toward Tregs and thus, are not only helpful in preventing graft vs. host reaction of normal tissues, but also prevent miscarriage, and as mentioned, aid in immune tolerance to cancer cells [36].

### 3.3. Innate Lymphoid Cells (ILCs) in the Decidua of the Placenta and Cancer Cells

Uterine natural killer (UNK) cells, though having a similar lineage to circulating conventional NK cells, have certain unique features. These cells can become cytotoxic, similar to NK cells in the peripheral circulation, but seem to be important in the process of immune tolerance by release of certain cytokines that help proliferation, invasion, and avoidance of immune rejection [37].

NK cells are also a major constituent of the innate lymphoid cells in tumor tissue [38]. The CD56+, CD3- NK cells in tumor tissue help regulate adaptive T-cell response, and thus inhibit tumor infiltrating lymphocytes (TILs) [39,40,41].

## 4. Progesterone Associated Molecules That the Cytoplasmic 34 kDa Splice Variant of PIBF Plays a Role in Fetal-Placental and Malignant Tumor Proliferation and Metastases

### 4.1. The Importance of Progesterone (P) to Prevent Miscarriage

The importance of P in allowing a pregnancy to proceed to term has been known for almost 100 years [42]. The mechanism requires interaction of P with the P receptor. Blocking the P receptor by a P antagonist leads to termination of the pregnancy [43,44].

Thus, it seemed that a good target for cancer immunotherapy would be a common protein enzyme or some other molecule that is needed for immune evasion by both the fetal-placental unit and the malignant tumor yet is not needed by the human once born. The lead author found the monograph written by Dr. Julia Szekeres-Bartho entitled “Immunosuppression by progesterone in pregnancy” (copywrite 1992) introducing a protein that she referred to as the progesterone induced blocking factor could be that ideal protein to target for cancer immunotherapy (Immunosuppression by Progesterone in Pregnancy; Julia Szekeres-Bartho, CRC Press, Inc., Boca Raton, FL, USA 1992).

There is evidence that P interaction with membrane P receptors may produce immunomodulatory proteins, e.g., the progesterone induced blocking factor (PIBF) which may be needed by the fetal-placental unit to aid in proliferation, invasion, and possibly most important, evasion of immune surveillance, by controlling the cell population and subsequent cytokine production in favor of immune suppression [45].

### 4.2. The Progesterone Induced Blocking Factor (Alternate Name Progesterone Immunomodulatory Binding Factor 1)

As mentioned above the presence of the interaction of P with the P receptor is not essential for life, as evidenced by males, menopausal women, and anovulatory women, where serum P levels are low. It was hypothesized that if the similarities between cancer cells and the fetal-placental unit include the need for the P receptor, then the P receptor could be an ideal target for an anticancer drug to provide not only efficacy in inhibiting cancer growth, but also with a high safety profile [45,46]. The hypothetical model considered the PIBF protein as a potential ideal target that may be needed for cancer progression, but not needed for normal human or animal function [47].

PIBF complementary DNA encodes a protein composed of 757 amino acids, with a predicted molecular mass of 89–90 kDa [19]. The 48 kDa N terminal port is biologically active [48]. The PIBF gene has been located on chromosome 13 [48]. PIBF shows no significant amino acid sequence homology with any known protein [48]. This 757 amino acid protein, or parent form, has a centrosomal position in the nucleus [49]. The mRNA transcribed from the PIBF1 gene contains 18 exons and codes for this 89–90 kDa protein [48]. The full length 89 kDa PIBF protein plays a role in cell cycle regulation [49,50,51]. There is evidence that the parent form of PIBF is the same protein as CEP90 a pericentriolar satellite protein that is crucial for integrity of the mitotic spindle [52]. Cell cycle regulation plays an important role in regulation of the invasiveness of both the trophoblast and malignant tumors [53,54,55].

The parent form of PIBF is converted to shorter cytoplasmic splice variants that have immunosuppressive activity. A 34 kDa splice variant produced by gamma/delta T-cells (and possibly cells of the fetal-placental unit, i.e., mesenchymal cells, embryonic cells, and trophoblast cells) cause a shift toward TH2 dominance favoring immunosuppressive cytokines, e.g., interleukin (IL)-10, IL-4, IL-5, and IL-6 [56,57,58]. These splice variants are located in the cytoplasm [49].

The smaller splice variants are secreted and bind to the GP1 anchored PIBF receptor [59]. This binding to the PIBF receptor forms a heterocomplex with the alpha chain of the IL-4 receptor [59]. This is at least partially responsible for the switch from TH1 to TH2 dominance during a viable pregnancy [59]. It should be noted that the PIBF receptor does not have a transmembrane association with the alpha chain of the IL-4 receptor so that it signals through the JAK/STAT pathway [59].

Decidual NK cells represent about 25% of cell decidual lymphocytes in the first trimester of pregnancy [60]. There is evidence that PIBF helps to stabilize perforin granules and granzymes A and B, and thus, inhibits the release of these cytotoxic substances [61,62].

### 4.3. Evidence That the Cytoplasmic 34 kDa Splice Variant of PIBF Plays a Role in Preventing Immune Rejection of the Fetal-Placental Unit

There is little question that the secretion of P is essential for the maintenance of a normal pregnancy. Taking a P receptor antagonist, e.g., mifepristone, even for just one day can terminate a normal pregnancy. When P binds to its receptor it induces structural alterations that enable DNA binding and the induction of genes resulting in the production of various proteins that may help the trophoblast to proliferate, invade normal tissue, and evade immune surveillance [46,63,64]. There is evidence that PIBF is one of these proteins that allows the fetal-placental unit to grow and avoid immune rejection [65,66,67,68,69,70,71,72,73,74,75,76,77].

### 4.4. Cancer Cell Line Studies to Evaluate Whether PIBF Is Secreted by Cancer Cells and the Effect of P and Anti-PR Drugs on PIBF up and Downregulation

In 2004, Lachman et al. published data showing PIBF to be present in the cells and tissues of several different types of cancers [49]. They found that PIBF was overexpressed in solid tumors from the stomach and uterus. It has been found in cancer cells from the ovary, cervix, breast, and lymphoma and leukemia cells [49,78]. In 2007, a study was published evaluating multiple different human leukemia cell lines, and PIBF secretion by these cells [78]. One interesting finding was that there was by far more mRNA dedicated to the production of the 34–35 kDa immunomodulatory protein form of PIBF than mRNA for any other protein made by the leukemia cells. Adding P to the media upregulated PIBF mRNA levels and increased secretion of the PIBF protein, whereas the P receptor modulator mifepristone suppressed PIBF mRNA decreased secretion of the PIBF protein itself [78].

A 57 kDa splice variant of PIBF was detected in glioblastoma multiforme cell lines [79]. Similar to the study of leukemia cell lines, adding P to the media upregulated the 57 kDa PIBF protein [80]. Furthermore, adding PIBF protein to the media increased the number of U87 cancer cells on days 4 and 5 of treatment [80].

It is clear that cancer cells do not secrete anywhere near the amount of P that is made by the fetal-placental unit. Thus, one may ponder how the tumor cells can stimulate PIBF secretion? One possible mechanism is that cancer cells develop the ability to make P, which increases PIBF in the tumor microenvironment, but is insufficient to be detected in the plasma. One study found that a large variety of cancer cells can express the human chorionic gonadotropin (hCG) beta subunit gene [81]. Production of hCG could lead to local P production, which may interact with P receptors in the cancer cells and could lead to local PIBF production. The PIBF would be made only in the tumor microenvironment, and thus would not increase PIBF plasma levels [82,83].

## 5. Animal and Clinical Trials Using Mifepristone to Inhibit Cancer Progression

### 5.1. Early Human Clinical Trials with Cancers Positive for the Classical Nuclear P Receptor

With the demonstration of classical nuclear progesterone receptor (nPRs) in some cancers, e.g., breast, ovarian, and endometrial cancers, the hope was that the nPR was essential for cancer growth, so that by blocking the nPR cancer, progression could be halted. Unfortunately, the clinical outcome with P receptor antagonist therapy was disappointing [84,85,86,87,88,89,90,91]. Thus, enthusiasm for treating some cancers with mifepristone waned.

### 5.2. Controlled Studies Evaluating Efficacy of Mifepristone in Improving Quality and Length of Life in Spontaneous Murine Cancers Not Known to Be Associated with the Classical nPR

Based on the demonstration that mifepristone suppressed PIBF expression by leukemia cell lines, the first group of mice was gavaged with the equivalent of the 200 mg daily dosage in humans. They showed improved longevity and quality of life (as evidenced by body conditioning scores) in aldo-keto reductase/J mice with spontaneous leukemia [92].

A/J mice, with spontaneous lung cancer, were the next group tested. The data showed that more than twice as many mice treated by mifepristone survived (57.6% vs. 27%). Even more impressive, the mean number of sick days (BCS < 4) for the mifepristone treated mice was only 11.6 days vs. 57.6 days for the controls [93].

Even though sometimes in humans nPRs have been found in some prostate and testicular cancers, the next groups of mice tested were C57BL/6 mice with prostate cancer and P3J/J mice with testicular cancer (even though the nPR was not present in these mice). They were chosen to see if cancers that are only present in males, could also respond to mifepristone and show a positive therapeutic effect [94]. Mifepristone also provided increased length and quality of life in these mice [94].

### 5.3. Human Experience in Treating Various Advanced Cancers Not Associated with the Classical nPR with Mifepristone

Related to the sensitivity of many people around the world to the subject of therapeutic abortion, most countries made laws making it difficult for physicians to prescribe the 200 mg dosage of mifepristone, which had been approved as an abortifacient [44]. In the United States, only licensed doctors approved to perform therapeutic abortions are granted permission to use the drug.

However, based on cancer cell line studies and controlled animal studies, the United States Food and Drug Association has granted off-label use of oral mifepristone 200 mg/day to treat end-stage cancer patients, who have exhausted all conventional therapies. For each case, it was required to apply to the FDA for a compassionate use investigational new drug approval (IND).

One exception was a man with multifocal renal cell carcinoma, who was not very advanced as yet. However, at that time there were no drugs to treat renal cell carcinoma and the patient wanted to avoid hemodialysis, which would have become necessary if he followed his oncologist’s suggestion for bilateral nephrectomy. Thus, he had a laparoscopic hemi-nephrectomy of his right kidney, leaving his left kidney, with three lesions present. For over 10 years, mifepristone kept these lesions from growing and no new lesions appeared. Eventually, his diabetes caused him to develop kidney failure and he then had a bilateral nephrectomy (after 11 years of mifepristone therapy). He was subsequently approved for a kidney transplant, and he is still cancer free and healthy 19 years after initial treatment with mifepristone [95].

Similar to the significant clinical benefits of mifepristone treatment for mice with lung cancer, there have been several case reports of patients with advanced metastatic lung cancer with no more treatment options, who have demonstrated marked improvement following treatment with oral mifepristone (some with 200 mg/day and some 300 mg/day), as evidenced by marked improvement in quality of life and significant extension of length of life [96]. One woman with chronic lymphocytic leukemia developed a probable small cell lung cancer with the syndrome of inappropriate anti-diuretic hormone (SIADH) syndrome. Her multiple lung lesions disappeared shortly after single agent mifepristone, leaving her only with a ground glass appearance to the lungs on chest X-ray. She remained in total remission for 5 years when she died at age 83, with a myocardial infarction [97,98]. It is generally considered that when lung cancer is associated with SIADH, death is usually imminent.

Similarly, some cases of non-small cell lung cancer (NSCLC) have shown marked improvement in quality and length of life, following single agent mifepristone therapy. One man with chronic obstructive lung disease developed stage IV NSCLC with brain metastasis, despite standard chemotherapy. With single agent mifepristone, he enjoyed 5 more years of life with no more evidence of any brain metastasis following his initial radiotherapy, and his lung lesions were stable then grew very slowly. When he died of pneumonia at age 73, he still had a very high quality of life, and as yet his lung cancer, per se, was not causing him any significant morbidity (unless the lung cancer contributed to him developing pneumonia, the actual cause of his death) [99]. For compassionate use the dosage of mifepristone is 200 mg daily. In his case he took 300 mg daily since he was the first case of an FDA approved investigator-initiated study using mifepristone 300 mg daily for stage IV NSCLC that had progressed despite two courses of chemotherapy or immunotherapy.

The aforementioned man with NSCLC did not have any tumor markers to target. Single agent mifepristone has, however, also provided significant improved quality and length of life in a patient with NCSLC who was positive for PD-1, who had progressed to stage IV, despite chemotherapy and the checkpoint inhibitor nivolumab [100]. She was the second patient enrolled in the investigator-initiated study. Unfortunately, she was the last patient treated in this study. The study had been approved for 40 patients. It was stopped for lack of enrollment not for disappointing results. Similarly, single agent mifepristone has provided marked palliative benefits and extension of life for two women with NSCLC, with brain metastasis, who were positive for the epidermal growth factor receptor (EGFR) mutation, who progressed despite treatment with the third-generation tyrosine kinase inhibitor osimertinib [101]. They are still alive after four years of 200 mg daily mifepristone therapy and doing well.

Mifepristone appears to cross the blood–brain barrier, not only as evidenced by the aforementioned cases of NSCLC with brain metastases, where no progression was found despite years of treatment, but also in a case of glioblastoma multiforme stage IV [99,101,102].

Mifepristone has provided palliative benefits leading to significant life extension in some moribund patients, including pancreatic cancer and stage IV colon cancer [103,104,105]. Other patients, with a variety of different cancers, have shown palliative benefits to mifepristone treatment when no other treatment options were available, including thymic epithelial cell carcinoma, transitional cell carcinoma of the renal pelvis, leiomyosarcoma, malignant fibrous histiocytoma, and fibroblastic osteogenic sarcoma [105,106,107].

The large majority of patients with advanced cancer treated with mifepristone have shown palliative benefits [95,96,97,98,99,100,101,102,103,104,105,106]. There have been a minority of cases where mifepristone was started days before death. Nevertheless, it is the hope that publishing these case reports will generate interest in some oncologists who treat a large cancer population to help determine the efficacy of mifepristone therapy in a larger series whether randomized or compared to known expectations.

It should be noted that since serum levels of PIBF do not decrease in patients with cancer, we can only speculate that the benefit of mifepristone may have been related to decreasing PIBF levels in the tumor microenvironment since this measurement would not be possible in a living human being [107].

### 5.4. The Possibility That the Benefits of Mifepristone Treatment for Cancer May Involve Some Protein or Pathway Other Than PIBF

Another protein that increases cancer virulence is known as the progesterone receptor membrane component-1 (PGRMC-1), protein. PGRMC may enable small amounts of P secreted to magnify its effect on membrane P receptors, which may increase speed of proliferation of cancer cells, or their ability to invade normal tissue [108,109]. Upregulated PGRMC-1 protein expression and mRNA in malignant tissues, has been found to correlate with poor overall survival, poor quality of life, and decreased tumor-free interval related to increased metastases and tumor size [110,111,112].

Against the hypothesis that the main beneficial effect of mifepristone in inhibiting cancer progression, is by suppressing PGRMC-1 (which in high dosages does suppress PGRMC-1 in cell line studies), is the fact that the lower dosage of mifepristone (200–300 mg) daily, which shows definite clinical benefits in patients with cancer, acts as an agonist, rather than antagonist, for PGRMC-1, and thus may increase cancer aggressiveness [113].

Thus, one could hypothesize that the efficacy of P receptor antagonist therapy could be increased by developing a P receptor modulator that either acts neutral, or even suppresses PGRMC-1 in lower dosages. Another option would be to develop a pure P receptor antagonist with no anti-glucocorticoid receptor activity, so that high dosages of this PR antagonist could be prescribed without causing adrenal insufficiency. Alternatively, one could concomitantly add a drug that inhibits PGRMC-1, e.g., AG-205 [114].

## 6. Conclusions and Final Thoughts

The ideal anticancer therapy would be one that could target a molecule that is essential for the cancer to thrive, but not needed for normal human function. This ideal targeted molecule would have an even greater value if this was required, not just by a minority of cancers, but for most cancers to proliferate.

Looking at the cancer as a foreign transplant has led to some therapies using autologous tumor vaccines [46]. Even more efficacious is targeting check-point inhibitors that help the malignant tumor with its neoantigens, to escape immune surveillance, but are also needed for the body to prevent attack of normal organs [27,28,29]. Unfortunately, though there are more than one type of tumor that relies on check-point inhibitors, only a minority of a given type of tumor seem to require check-point inhibitors to escape immune surveillance [27]. Nevertheless, treatment of several cancers with anti-PD-1 drugs, e.g., nivolumab, or pembrolizumab, has provided an extension of a better quality of life in people with certain malignancies [27]. However, eventually the malignant tumor finds a way to mutate, so it no longer needs that molecular pathway to proliferate and develops resistance to check-point inhibitors. Furthermore, because check-point inhibitors are needed to prevent autoimmune reaction against normal tissue, these drugs do present risk of autoimmune disorders (but generally less severe side effects than chemotherapy).

As mentioned, the monograph by Julia Szkeres-Bartho published in 1992, describing her research on the PIBF protein, provided the impetus to perform our studies in cancer to see if this could be the ideal molecule to target for cancer therapy. Dr. Julia Szekeres-Bartho has referred to PIBF as the “double-edge sword” in that, whereas it is essential to bring in life by allowing the feto-placental unit to thrive, it seems to be also responsible for death by cancer, which also uses this protein to help the tumors to proliferate and evade immune surveillance [115].

Unfortunately, testing tumors, such as Foundation 1 testing for specific markers, does not include the PIBF marker, but based on the wide variety of cancers that seem to respond to P receptor antagonists, e.g., mifepristone, the data suggest that PIBF may be required by most tumors to thrive. Thus, it might be helpful to have PIBF included in tumor marker testing.

Empirical therapy with P, without specific evidence of a P deficiency, has led to marked correction of infertility issues and the prevention of miscarriage [22,116,117]. There is evidence that P alone can increase the secretion of PIBF from circulating gamma/delta T-cells [118]. However, if there is an excessive amount of NK cells in the fetal microenvironment, more PIBF may be needed to prevent these cells that are required to enable uterine artery remodeling to create spiral arteries, to then attack the fetal semi-allograft [119]. Similarly, the PIBF used by the malignant tumor to escape immune surveillance may be mostly required in the tumor microenvironment. Thus, empirical use of anti-progesterone drugs may be a reasonable approach to treating cancer, even if one cannot perform tests to determine if suppressing PIBF is necessary.

Based on its efficacy in improving quality of life and increased longevity in patients with a variety of different advanced cancers (even when all other therapies have failed), its lack of side effects, convenient oral method of administration, relative low cost, mifepristone may be the best single agent anti-cancer treatment available today for treating advanced cancers [120]. Unfortunately, because this treatment is not being promulgated by the pharmaceutical manufacturers for off-label use, most clinical oncologists are not familiar with this treatment, or are only aware of early studies with cancers associated with the classical nPR, that were disappointing. The possibility exists that the disappointing results with mifepristone therapy for cancers positive for the nPR, could be related to the fact that in the presence of the nPR these cancers are generally associated with a better prognosis [107]. The use of mifepristone for breast and ovarian cancer, while suppressing the PIBF protein, may also negate the role that the nPR plays in inhibiting possibly some other protein, e.g., hypothetically, PGRMC-1, that promotes tumor proliferation. 

It is also possible that in the early days tumor regression was the main endpoint of a study of cancer therapy but these controlled animal studies and human experience indicates that perhaps there should be a shift in main endpoints to establish treatment benefit to increased longevity and improved quality of life.

Hopefully, continued publications related to the efficacy of mifepristone for treating various cancers will generate interest in some oncologists to initiate larger clinical trials or generate interest in some pharmaceutical companies to try to develop and patent even more efficacious P receptor modulators, or possibly explore the use of monoclonal antibody therapy directed against PIBF. It is possible that the beneficial effects of the P receptor antagonist, mifepristone, in patients with cancer, though initiated initially with the concept of inhibiting PIBF, could be found following future studies to be related to inhibiting some other substance than the PIBF protein. However, response to a monoclonal antibody against PIBF would greatly support the hypothesis that the main benefit of mifepristone treatment in inhibiting cancer progression is via suppressing the PIBF protein.

**Figure 1:**
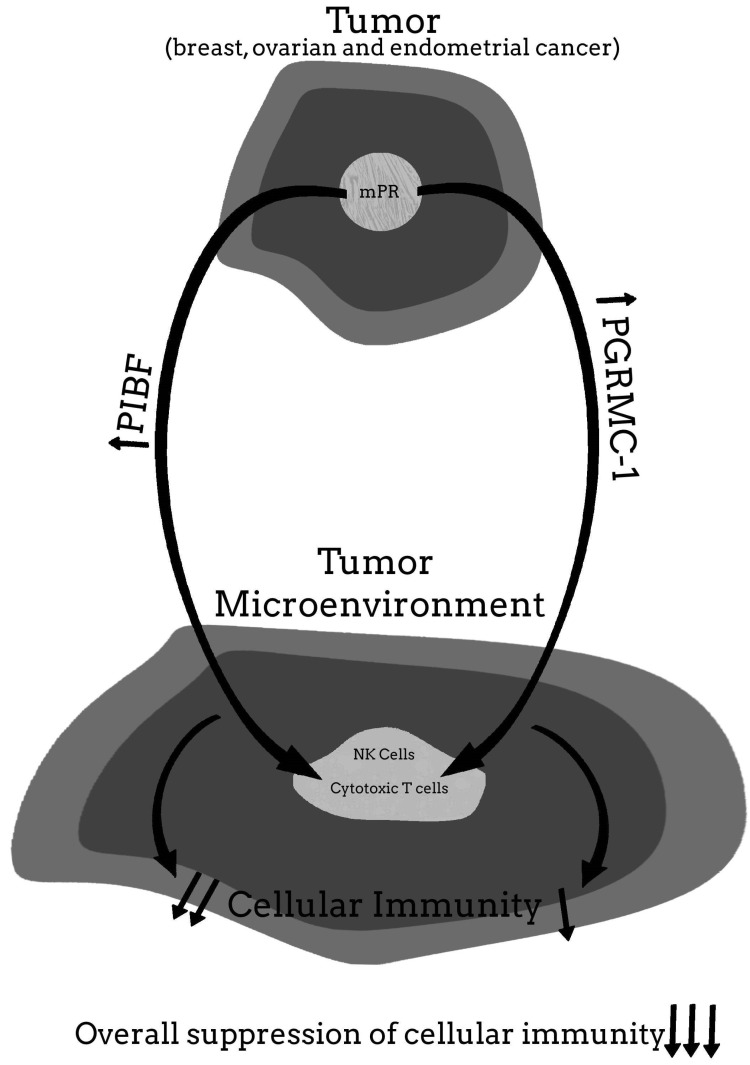
Malignant tumor without nuclear progesterone receptor (nPR) but membrane PR (mPR) present.

**Figure 2:**
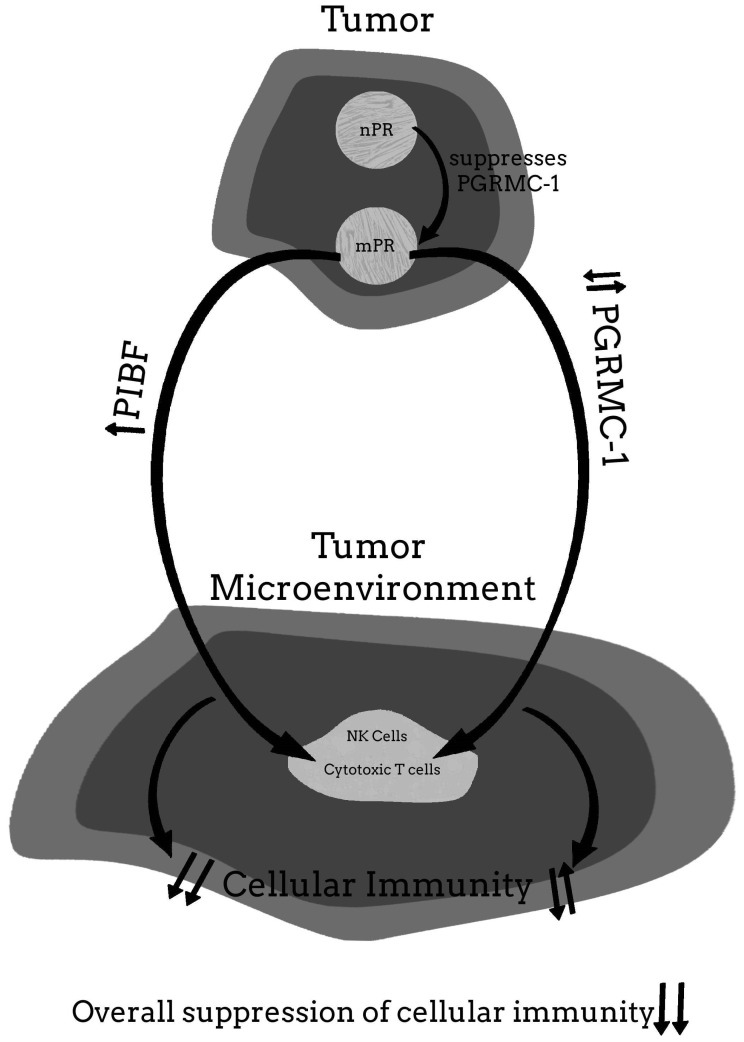
Malignant tumor with nuclear progesterone receptor (nPR) and membrane PR (mPR).

**Figure 3:**
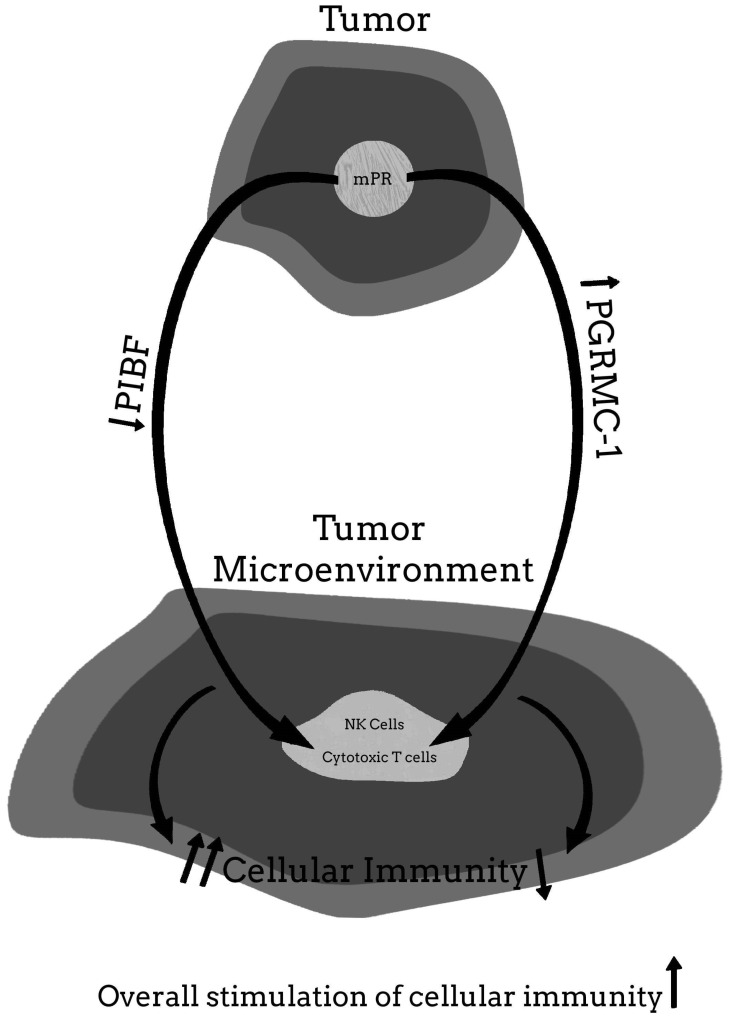
Malignant tumor without nuclear progesterone receptor (nPR) but membrane PR (mPR) present—with low dose mifepristone therapy.

**Figure 4:**
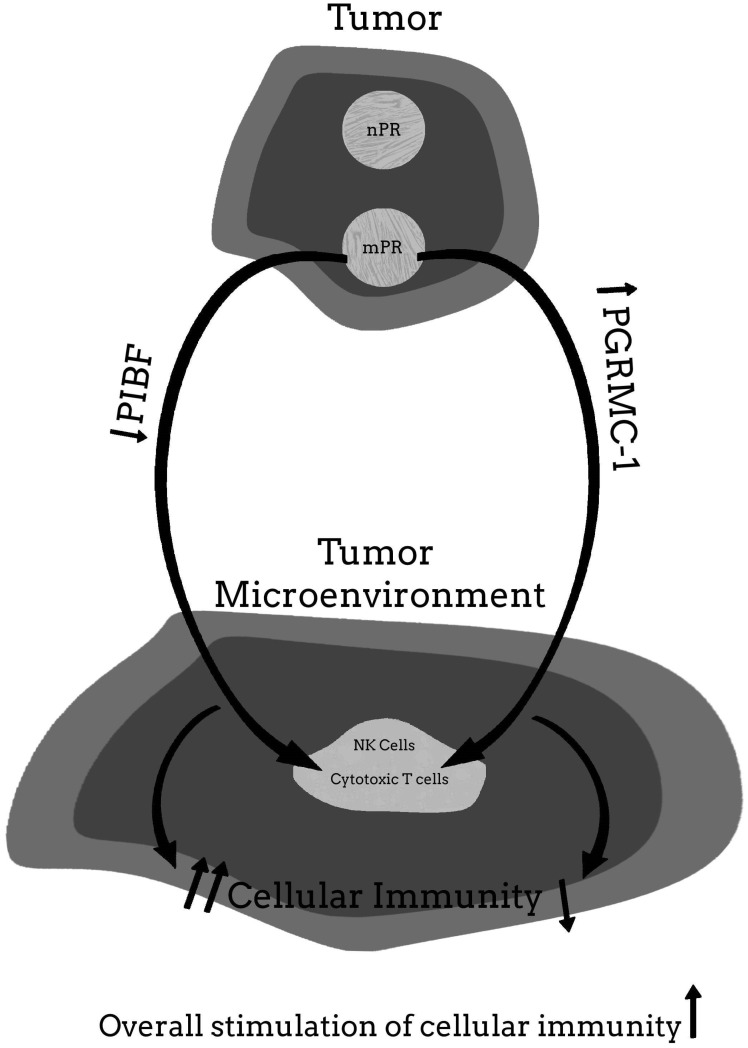
Malignant tumor with nuclear progesterone receptor (nPR) and membrane PR (mPR) present—with low dose mifepristone therapy.

**Figure 5:**
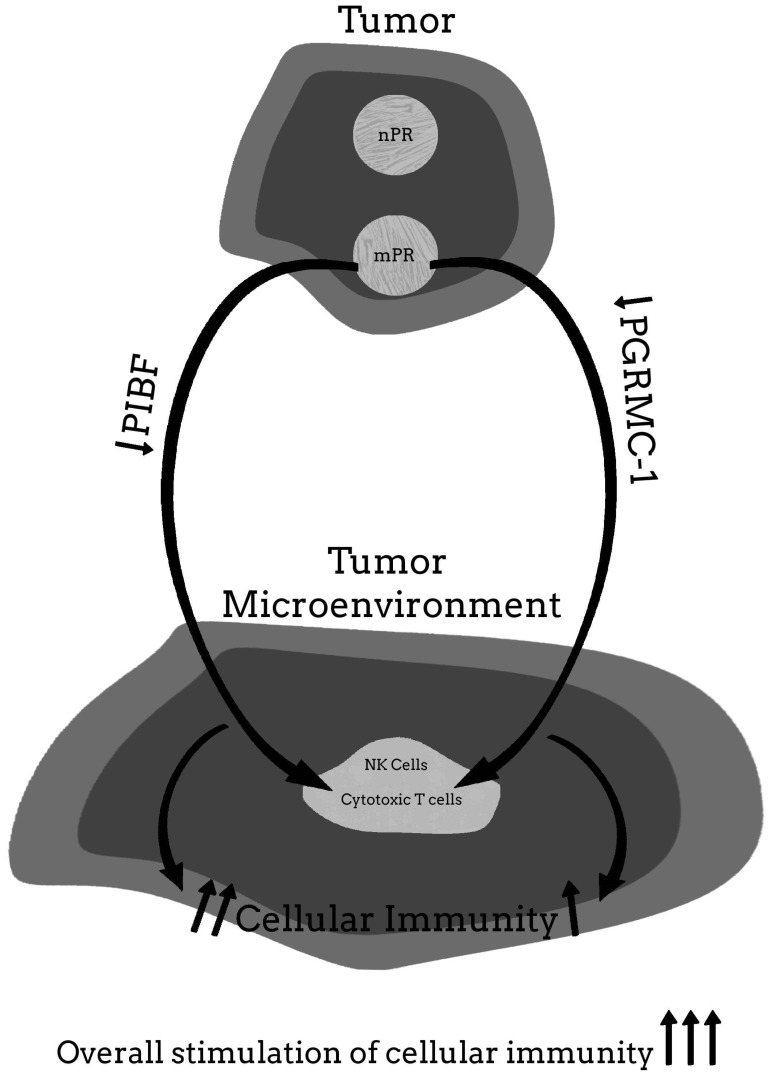
Malignant tumor with or without nuclear progesterone receptor (nPR) present—using high dose PR antagonist devoid of glucocorticoid receptor antagonist activity (not developed as yet).

For more clarity on the proposed hypothetical model of how the PR may be involved in cancer proliferation, and how blocking the PR may help to provide significant clinical relief from the effects of cancer, Figure 1, Figure 2, Figure 3, Figure 4 and Figure 5 provide a visual mechanism of the hypothetical ways that there may be interaction of the nuclear and membrane PRs and the production of immunomodulatory proteins. Furthermore, these figures will show the hypothetical mechanism involved in the anti-cancer benefits of PR antagonists.
